# One-Week Suicide Risk Prediction Using Real-Time Smartphone Monitoring: Prospective Cohort Study

**DOI:** 10.2196/43719

**Published:** 2023-09-01

**Authors:** Maria Luisa Barrigon, Lorena Romero-Medrano, Pablo Moreno-Muñoz, Alejandro Porras-Segovia, Jorge Lopez-Castroman, Philippe Courtet, Antonio Artés-Rodríguez, Enrique Baca-Garcia

**Affiliations:** 1 Department of Psychiatry Jimenez Diaz Foundation University Hospital Madrid Spain; 2 Institute of Psychiatry and Mental Health Hospital General Universitario Gregorio Marañón Madrid Spain; 3 Department of Signal Theory and Communications Universidad Carlos III de Madrid Madrid Spain; 4 Evidence-Based Behavior (eB2) Madrid Spain; 5 Cognitive Systems Section Technical University of Denmark Lyngby Denmark; 6 Department of Psychiatry Centre Hospitalier Universitaire Nîmes Nîmes France; 7 Institut de Génomique Fonctionnelle CNRS-INSERM University of Montpellier Montpellier France; 8 Department of Emergency Psychiatry and Acute Care Centre Hospitalier Universitaire Montpellier France; 9 Centro de Investigación Biomédica en Red de Salud Mental (CIBERSAM), Carlos III Institute of Health Madrid Spain; 10 Instituto de Investigacion Sanitaria Gregorio Marañón Madrid Spain; 11 Department of Psychiatry Autonomous University of Madrid Madrid Spain; 12 Department of Psychiatry Rey Juan Carlos University Hospital Móstoles Madrid Spain; 13 Department of Psychiatry General Hospital of Villalba Madrid Spain; 14 Department of Psychiatry Infanta Elena University Hospital Valdemoro Madrid Spain; 15 Department of Psychology, Universidad Catolica del Maule Talca Chile

**Keywords:** e-health, m-health, Ecological Mometary Asssessment, risk prediction, sensor monitoring, suicidal, suicide attempt, suicide

## Abstract

**Background:**

Suicide is a major global public health issue that is becoming increasingly common despite preventive efforts. Though current methods for predicting suicide risk are not sufficiently accurate, technological advances provide invaluable tools with which we may evolve toward a personalized, predictive approach.

**Objective:**

We aim to predict the short-term (1-week) risk of suicide by identifying changes in behavioral patterns characterized through real-time smartphone monitoring in a cohort of patients with suicidal ideation.

**Methods:**

We recruited 225 patients between February 2018 and March 2020 with a history of suicidal thoughts and behavior as part of the multicenter SmartCrisis study. Throughout 6 months of follow-up, we collected information on the risk of suicide or mental health crises. All participants underwent voluntary passive monitoring using data generated by their own smartphones, including distance walked and steps taken, time spent at home, and app usage. The algorithm constructs daily activity profiles for each patient according to these data and detects changes in the distribution of these profiles over time. Such changes are considered critical periods, and their relationship with suicide-risk events was tested.

**Results:**

During follow-up, 18 (8%) participants attempted suicide, and 14 (6.2%) presented to the emergency department for psychiatric care. The behavioral changes identified by the algorithm predicted suicide risk in a time frame of 1 week with an area under the curve of 0.78, indicating good accuracy.

**Conclusions:**

We describe an innovative method to identify mental health crises based on passively collected information from patients’ smartphones. This technology could be applied to homogeneous groups of patients to identify different types of crises.

## Introduction

Each year, suicide is the cause of about 1.4% of all deaths worldwide, totaling approximately 800,000 lives lost, which means that more people die from suicide than from war and homicide combined [1]. In 2020, the first full year of the COVID-19 pandemic, suicide was responsible for nearly as many years of potential life lost as the disease [2], making death by suicide a major public health issue worldwide and one that is worsening in spite of preventive efforts [3].

Interventions aimed at reducing the risk of suicide rely on the effective identification of high-risk patients. There are many known risk factors underlying suicide mortality, though their ability to identify people at risk for suicide is scarcely better than chance [4]. Furthermore, traditionally known factors such as younger age, depression, and history of childhood trauma are useful in predicting suicide in the long term (months or years), though not over a period of weeks or days [5-7]. Therefore, prevention of suicide urgently requires improved short-term prediction methods [8].

Nowadays, the gold standard method of assessing suicide risk is the clinical interview, which, though clearly more effective than questionnaires [9,10], is not without its limitations. Specifically, data obtained in the course of a clinical assessment are cross-sectional and, as such, are useful only for a brief period [11]. Additionally, categorizations of suicide risk based on clinical findings frequently result in a high false positive rate (FPR) while overlooking many deaths owing to suicide [12]. In response to this, machine learning approaches have been designed in recent years which hold promise as an improved method of predicting mental health crises in general [13] and suicide risk in particular [14]. Regarding mental health crises, a recent work [13] predicted critical events within 1 month by using a machine learning model that analyzed data from electronic health records, achieving an area under the curve (AUC) of 0.797.

There is a pressing need to move away from methods of suicide prediction based on risk stratification in favor of approaches that use individual risk measures [15]. Though suicide is a public health problem of the highest order, an individual approach is required to decrease suicide, and to do so, a precision health perspective may be useful [16]. Technological advances widely available for consumer use provide an ideal setting to advance toward preventive medicine by harnessing the personalized data they generate. As technology becomes increasingly embedded in our lives, people’s digital devices (eg, smartphones and wearables) produce a massive amount of data with possible clinical relevance. This information, together with patient and caregiver self-reported data, is referred to as “patient‐generated health data” [17] and has proven to be useful in fields such as oncology [18], ambulatory cardiac monitoring [19], and neuropsychiatric illnesses [20]. Recently, the term “digital phenotyping” was coined, referring to descriptions of behavior based on people’s interaction with their smartphones (eg, sensor information, keyboard interaction, voice, and speech data) [21]. The data that make up a person’s digital phenotype may be either active (requiring input from the user, collected through Ecological Momentary Assessment [EMA]) [22] or passive (gathered by sensors incorporated in the device, requiring no action by the user) [23].

In the field of suicide prevention, only active data have been widely used to depict the suicidal digital phenotype [24] and combining active data with information from electronic health records has been demonstrated to be useful in predicting suicide [25]. In contrast, although its feasibility has been demonstrated in acute mental health setting [26], no studies to date have proven the predictive potential of passive data. Given the low rates of compliance with EMA among patients with suicidal ideation [24], there is great interest in such automatically generated data in the field of suicide prevention and research.

In this study, we aimed to test the effectiveness of a smartphone-based system for continuous monitoring of patients with suicidal ideation. We used passively collected data from embedded smartphone sensors to detect behavioral changes before a clinical risk event in patients participating in the Spanish branch of the SmartCrisis Study [27], designed to study suicide risk factors in a cohort of patients with a history of suicide behavior or suicidal ideation. We hypothesized that our system would be able to detect changes in a window of 1 week before a risk event.

## Methods

### Overview

The SmartCrisis study [27] was conducted from February 2018 to March 2020 in Madrid (Spain) and Montpellier and Nimes (France). For this analysis, only data from Spain were used.

### Study Design and Sample

Participants were outpatients with any psychiatric diagnosis undergoing follow-up in the program for secondary suicide prevention at the Fundacion Jimenez Diaz Mental Health Department.

Inclusion criteria were age 18 years or older, a history of suicide behavior or suicidal ideation according to the Columbia Suicide Severity Rating Scale [28], ability to understand and sign the informed consent form, and ownership of a smartphone connected to a Wi-Fi network at least once a week. Patients were not compensated for their participation.

All participants downloaded the Evidence-Based Behavior (eB2) app to their smartphones. Developed by the Department of Signal Theory and Communications at Carlos III University in Madrid, the app collects data from smartphone sensors [29].

### Measures

Sociodemographic variables, including age, sex, marital status, and employment status, were collected. Psychiatric diagnoses were clinical, based on information contained in the electronic health record according to the criteria contained in the 10th edition of the International Classification of Diseases [30].

Participants were monitored for 6 months using their own smartphones and assessed at baseline, 6 months, and at the end of the study using a range of questionnaires. We also collected information on clinical status throughout the year using electronic health records, which enabled us to identify suicide attempts or visits to the emergency department requiring psychiatric assessment, which were our proxies for suicide risk (ie, risk events). We decided to broaden the scope of suicide risk status using this proxy given the relative infrequency of suicide attempts, as the rate of nonfatal attempts in the year following a previous attempt is only 16% to 17% [31,32], and given the relationship between emergency department visits and suicide [33]. For every participant, we considered the first event a risk event, and after that, the monitoring was stopped.

The eB2 app collects, merges, and preprocesses the following raw data: actigraphy, location tracking (ie, global positioning system [GPS]), device usage, and activity measured by Google Fit. These raw data generated by smartphone sensors were used to build half-hourly summaries of the location, steps taken, and distance walked, as well as app usage for the day. The output data representation was used to model patients’ daily routines, which we defined as daily profiles. A distribution of these profiles over a series of days constituted a behavioral pattern; changes between these behavioral patterns represented a potential crisis to be correlated with suicide risk.

### Ethical Considerations

The study was approved by the Fundacion Jimemez Diaz Research Ethics Committee (PIC 99/2017_FJD) and carried out in compliance with the tenets of the Declaration of Helsinki [34]. All patients gave written informed consent to participate after a complete description of the study, they were not compensated for their participation. Concerning data protection and confidentiality, each patient’s identification was ensured by a username and password. The data gathered by the eB2 app were anonymized if it were sensitive data, then translated into a unique data schema, and finally transmitted through Wi-Fi to the eB2 backend server where it were stored. The transmission was done through a RESTful application programming interface, which had been developed using the JAVASpring framework. This application programming interface is secure sockets layer protected, and, to restrict access to the patients’ information, a token-based access policy was implemented following the OAuth2 standard.

### Data Analysis

The system for data collection (eB2) is passive and unobtrusive. It is downloaded to participants’ own smartphones under the supervision of research assistants and works in the background, with no active user collaboration required. For this particular study, the app was configured to generate 4 types of variables from the raw smartphone data: distance traveled, time spent at home, steps taken, and use of any app. Each of these daily activity variables was processed from raw data as a 48-dimensional vector, with each component representing 30 minutes of activity. The “distance” variable is continuous and reflects the distance traveled by the patient, calculated as the difference between location traces. The “time spent at home” variable is considered binary: a value of 1 was assigned if the patient was at home at any time during a particular 30-minute slot and 0 if they were not. For each patient, the eB2 app identified participant’s home based on the processing of location traces. In particular, a patient’s home was considered as the place where he or she spends more time during nights (along time). The “steps” variable is continuous and contains the number of steps walked over a particular time slot. The “app usage” variable is binary, and a value of 1 was assigned if the patient used any smartphone app during the period and 0 for no app use. We used a logarithmic scale for the continuous variables of distance and steps.

**Figure 1:**
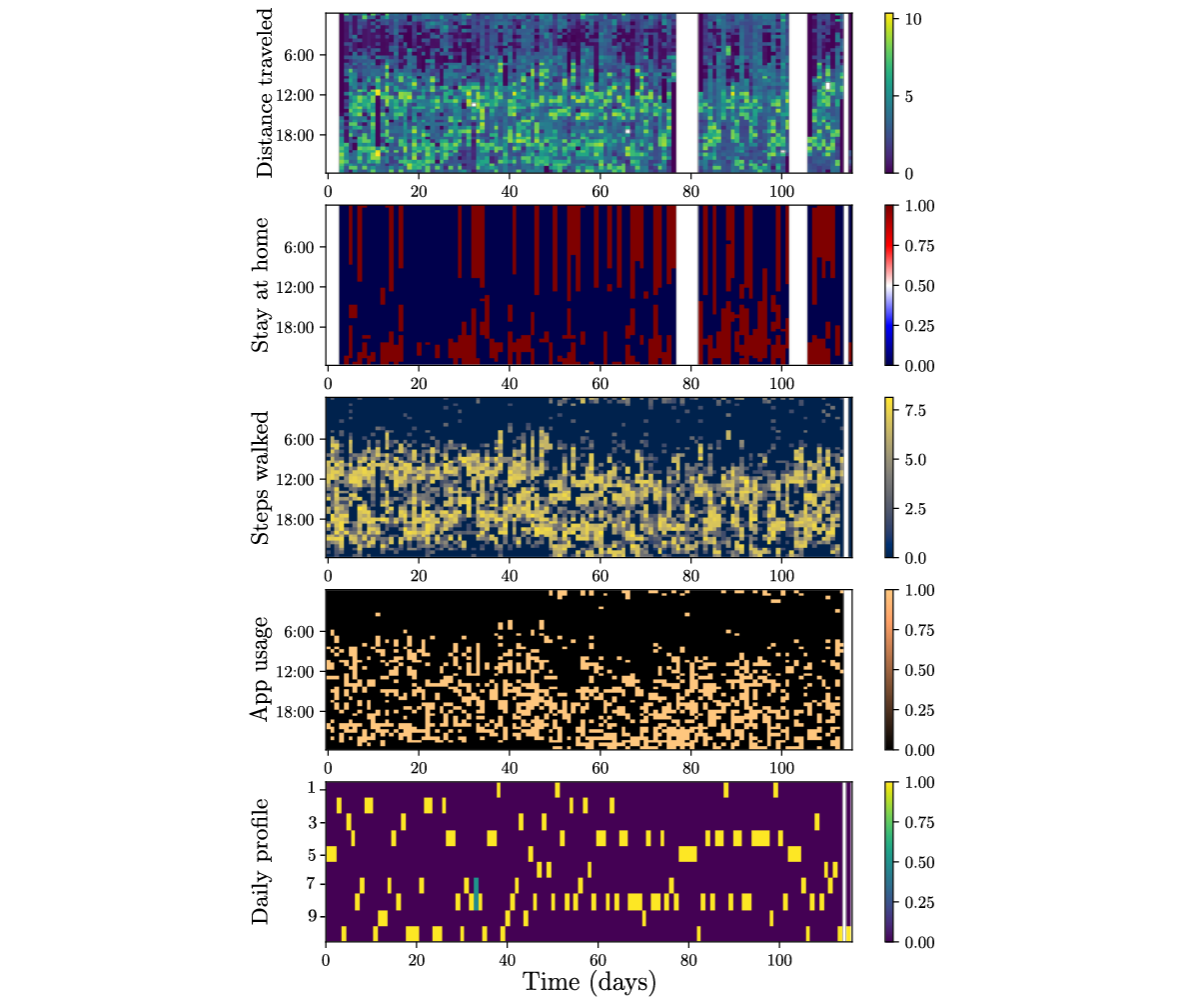
Daily observations after preprocessing and profile clustering assignment from a patient over 120 days. The x-axis is shared across the 5 graphics and represents time (in days). Four upper rows: distance traveled (logarithmic scale), time spent at home, steps taken (logarithmic scale), and app usage, respectively. For each figure, the y-axis indicates the value of the variable at each half-hour slot on the day. Fifth row: probability distribution over 10 possible daily profiles obtained for each day. The y-axis indicates the likelihood that a given day is described by each profile.

We created a model to obtain intuitive categorical variables representing patients’ profiles as reflected by data on mobility, physical activity, and app use (Figure 1). For instance, one value for the categorical variable could indicate a “high-activity” profile, with greater physical activity and more time spent outdoors, while another may indicate “low-activity,” with less physical activity and an increase in smartphone usage. We created a personalized model for each patient. We used an unsupervised model for this task, specifically a Heterogeneous Mixture Model with 10 components. The number of components defines the maximum number of possible profiles (10 in this case), where the final number of profiles for each patient is selected by means of a model selection method, the Bayesian information criterion. The details of the method can be found in the work by Moreno-Muñoz et al [35]. To simplify data management by machine learning models, the initial set of mobile data, which was high-dimensional (48 dimensions per variable) and composed of different data types (ie, binary and continuous), information corresponding to each day was represented by a 1-dimensional categorical variable indicating the daily profile.

In a second stage, we aimed to identify abrupt transitions in the generative distribution of daily profile sequences for individual patients. We defined each fixed distribution of profiles over a series of days as a behavioral pattern. An example of an abrupt transition would be when a patient with normally constant behavior consisting of 5 days per week of a “high-activity” profile alternating with 2 days of “low activity” shows a sudden inversion in this proportion (ie, 2 days of high activity and 5 days of physical inactivity). We consider these situations as change points, and we refer to them as behavioral changes, which we aimed to detect.

The change-point detection model used is based on the hierarchical extension of the Bayesian online change-point detection algorithm [36]. We assumed that the main sequence of daily profiles could be divided into nonoverlapping behavioral patterns separated by behavioral changes. We also assumed that the profiles were distributed unevenly within different behaviors, though the parameters of each distribution are unknown. The goal was to collect both the unknown parameters and the locations of behavioral changes. Since we cannot observe the true sequence of daily profiles representing a behavior, we must use the sequence of probability profiling vectors. The change-point detection method used was presented in our previous work [36] and uses samples of the posterior distributions to fully characterize the probability of each profile for each day. Using this method, a discrete variable is introduced into the model that counts the exact number of days since the last change-point occurred. The aim was to continuously infer the probability distribution of this variable given the data, obtaining a measure of uncertainty of the last location of behavioral change given the sequence of daily profiles up to that moment. Moreover, the proposed model is able to naturally integrate missing observations. Missing data are handled differently by the model when observations are partially missing (one or some features are totally or partially missing within a day) or totally missing (every feature is missing that day). The first case is handled in the daily profiling step through the Heterogeneous Mixture Model, following the approach detailed in [36,37]. The second situation is tackled in the second stage, when the change-point detection model is applied through the sequence of daily profile representations. In this case, we consider a Bayesian approach that is based on marginalization of missing observations, as also detailed in [36,37]. Treating missing data through algorithms instead of heuristic approaches provides robustness and allows to reduce the false alarm rate.

In a final stage, we defined a strategy to detect behavioral changes based on the cumulative probability that a change occurred over the previous days. Specifically, we consider that a behavioral change has been detected at day “d” if the cumulative probability that a change occurred over the previous 7 days exceeds a probability threshold that needs to be chosen a priori and we refer to as stability threshold. This point is in fact one of the model’s advantages because its choice provides flexibility to the detection mechanism, allowing the detection sensitivity to be adapted to the context of caregivers and patients. The lower the probability threshold, the greater the sensitivity for detection.

**Figure 2:**
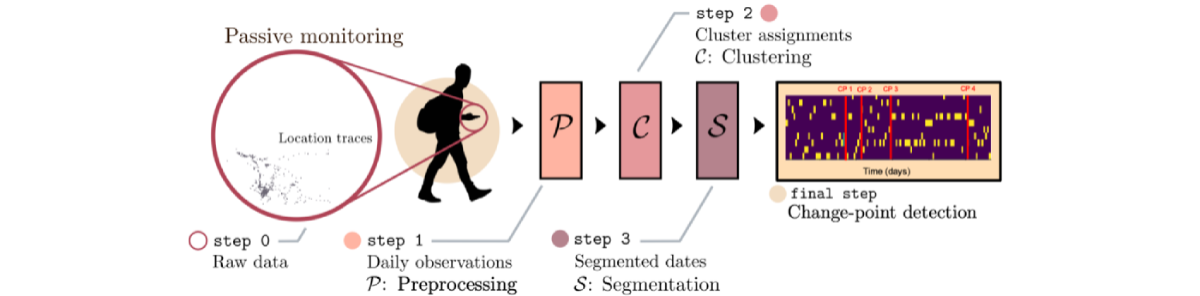
Diagram illustrating the collection, preprocessing, segmentation, and detection stages of the mobile health data collection system performed by the eB2 app.

The entire process is represented graphically in Figure 2.

In particular, the receiver operating characteristic curve has been generated over a range of values of this stability threshold. Specifically, we have applied the method for 50 values, equally distributed within the (0,1) interval, and for every patient in the sample. For each stability threshold, the FPR and true positive rate (TPR) have been calculated considering the predictions obtained for every patient, day, and risk event, resulting in as many data points as days we have in the whole cohort of patients. On a specific day where the method predicts an event that actually occurred, we consider that data point a true positive; on the other hand, if the method predicts an event that did not occur (usually called a false alarm), we consider that data point a false positive. Using a fixed stability threshold, these data points are used to compute the FPR and TPR, which are then represented as a point on the receiver operating characteristic curve.

**Figure 3:**
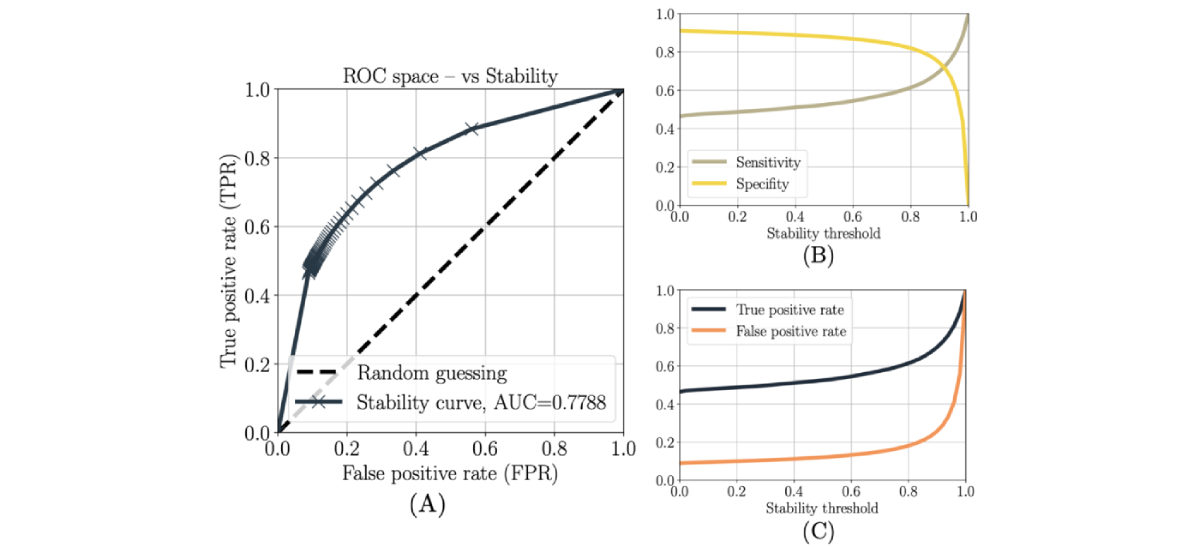
(A) Receiver operating characteristic curve of our detection method for predicting suicide-risk events in a 1-week window. This metric has been obtained over the stability threshold, defined as the probability threshold that considers whether a behavioral change occurred within the previous 7 days or not. We have applied our method for 50 values of the stability threshold and every patient in the sample. For each stability threshold, the false positive rate (FPR) and true positive rate (TPR) have been calculated considering the result obtained for every patient and suicide risk event. The cross points on the figure represent these 50 pairs (FPR and TPR) and the associated interpolation curve. (B) Model sensitivity and specificity for different values of the stability threshold. (C) TPR and FPR for different values of the stability threshold.

The cross points on Figure 3 represent these 50 pairs (FPR and TPR) for the 50 stability thresholds tested and the associated interpolation curve.

## Results

The sample consisted of 225 participants, 84 (37.3%) male and 141 (62.7%) female, with a mean age of 43.24 (SD 14.13) years. Among the total sample, most participants were diagnosed with mood or anxiety disorders. Sample characteristics are shown in Table 1.

**Figure 4:**
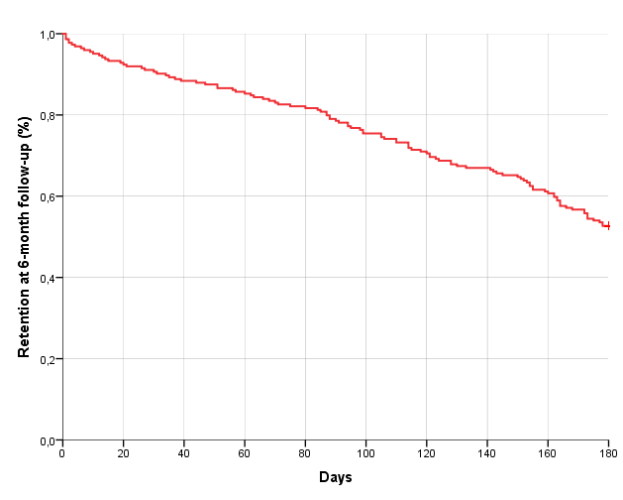
Survival curve of retention for the passive monitoring app over the 6-month follow-up period.

Participants were monitored by the eB2 app during 139.55 (SD 57.92) days, and at the end of the 6-month follow-up, 117 (52%) patients continued uploading data (Figure 4).

A total of 18 (8%) suicide attempts were recorded during the follow-up period, and there were 14 (6.2%) emergency department visits for psychiatric care; together, these totaled 32 (14.2%) suicide risk events.

The behavioral changes detected by the model described above predicted 1-week suicide risk with an AUC of 0.78 (Figure 3).

**Table 1:** Sample characteristics.

Characteristics		Values
**Gender, n (%)**		
	Male	84 (37.3)
	Female	141 (62.7)
Age (years), mean (SD)		43.24 (14.13)
**Marital status, n (%)**		
	Married or unmarried couple	77 (34.2)
	Single	90 (40)
	Separated or divorced	52 (23.1)
	Widowed	6 (2.7)
**Employment status, n (%)**		
	Employed or student	101 (44.9)
	Unemployed	50 (22.2)
	Retired	12 (5.3)
	Temporary leave	44 (19.6)
	Permanent leave	18 (8)
**Living with others, n (%)**		
	Yes	171 (76)
	No	43 (19.1)
**Psychiatric diagnosis, n (%)**		
	Mood disorders	104 (46.2)
	Anxiety disorders	169 (75.1)
	Personality disorders	109 (48.4)
	Drug abuse	24 (10.7)
	Eating disorders	10 (4.4)
	Psychotic disorders	1 (0.4)
**Suicidal history, n (%)**		
	Suicide attempt, previous year	94 (41.8)
	Suicide attempt, more than 1 year previously	85 (37.8)
	Lifetime suicidal ideation	46 (20.4)

## Discussion

Using smartphone-based monitoring, we followed up a cohort of patients at risk of suicide, identifying their behavioral profiles and changes to these profiles which predicted a period of clinical risk. Our results indicate that this innovative method based on passively collected information from patients’ smartphones is capable of detecting risk situations with high precision (AUC=0.78) over a 1-week period.

To date, many efforts have been made to classify the digital phenotype of patients with suicidal ideation, and a number of studies using smartphone-based EMA have reported high variability of suicidal thoughts [5,38-40] and have observed negative affect [41-46], environmental triggers [46,47], and altered sleep [48] preceding suicidal thoughts, though the ability of this methodology to predict risk of suicide has been less comprehensively explored. As an example, a recent study found that real-time changes in suicidal thoughts during psychiatric hospitalization predicted suicide attempts in the month after discharge with good accuracy (AUC=0.89) [25]. Passively collected data have been used in the mental health field to characterize stress and anxiety, depression, bipolar disorder, schizophrenia, and posttraumatic stress disorder [20], but no research has specifically studied suicidal behavior. In this study, we identified behavioral changes that alerted us to the risk of suicide or a mental health crisis over the following week.

Our results represent a breakthrough in personalized medicine to treat suicidal behavior. Our tool determines an individual patient’s risk status by comparing their present and past behavioral patterns and could be used to define suicide risk and design specific preventive interventions in line with prevention-oriented formulations [49]. Previous works have found that the ability to identify risk of suicide attempts after an emergency department visit improved with a machine learning prediction model that combines different sources of information: patient’s self-reports, information in the electronic health record, and clinician reports (1-month model: AUC=0.76; 6-month model: AUC=0.77) [50]. We theorize that if these sources were merged with passive data, a more accurate system for predicting suicide risk will be the result. This is of special relevance given evidence from an important meta-analysis reporting that 95% of patients classified as high-risk do not die by suicide, while half of all suicides occur in patients who were classified as low-risk [7].

We obtained a personalized short-term method of risk detection that can be adjusted for cost-effective intervention programs adapted to different health care systems. In low-resource systems, it may be optimal to select a threshold that preserves moderately high sensitivity and specificity; as a result, behavioral changes identified will likely correspond to clinical situations of suicide risk, and minor changes will not be misidentified. Therefore, the tool will not underdetect risk events and will not activate false alarms. In well-resourced health systems, where costlier suicide protocols can be implemented, a threshold can be selected to maximize sensitivity while accepting moderate to low specificity. Furthermore, given the accuracy obtained with our predictive model, adding a personalized intervention would be cost-effective as per recently proposed requirements [51].

Our model is remarkably robust in its handling of both total and partially missing observations. Both situations are naturally integrated into the model by assuming a Bayesian approach [36,37]. This is of special importance for clinicians who treat patients with an unclear risk of suicide, as clinical intuition might resemble missing data in at-risk situations.

Our app for smartphone-based monitoring in an outpatient sample of patients with a history of suicide illustrates the potential of passive monitoring techniques. We applied our tool to a large, clinically representative real-world sample with a range of diagnoses, following these individuals for an extended time period in a tax-funded health care system representative of most European contexts.

When research with passive data collection is made, ethical issues arise, and preserving privacy and confidentiality while simultaneously ensuring safety if suicidal risk is detected is a challenge [52]. Regarding this concern, we have to point out that in our specifical sample, monitoring is well accepted [53]. Furthermore, our ethics protocol complies with European standards.

In this study, some limitations should be acknowledged. First, in order to obtain a sufficient number of critical events, we decided to take into account not only suicide attempts but also any emergency room visits involving psychiatric care, as these have been found to significantly influence suicide risk [33]. Second, a number of patients were not under follow-up in our facilities after 6 months, which is inherent to our real-world design [53]. Third, we analyzed only a subset of data generated by smartphone-based monitoring of location traces, Google Fit activities, actigraphy, and app use logs. We focused on this set because of the quality of these data sources and the availability of the 4 sources for a large number of patients, thereby allowing us to increase the size of our sample. Future research could be enhanced by including data on light exposure, sleep, specific activities (eg, time spent playing sports), particular app usage (eg, messaging apps), and number of phone unlocks or calls, among others. However, these sources are either not available for every operating system or require a higher number of permissions from the user. Last, some patients willing to enroll in the study could not participate as they did not have a mobile phone or had a noncompatible device; this highlights the digital gap that researchers and clinicians must always account for when considering the inclusion of new technologies in health care. In fact, resource investment in digital protocols, which may be more cost-efficient in the long term, can optimize the use of traditional resources and narrow the digital gap.

Overall, the results of this cohort study indicate that an unsupervised machine learning approach to data obtained by passive real-time smartphone-based monitoring of patients with suicidal ideation is useful in predicting suicide risk. These data, when combined with actively collected data from patients and caregivers (ie, EMA), data from electronic health records, and clinical assessment, can improve suicide risk detection. Moreover, this technology may be transferable to other psychiatric conditions in which crisis prediction is needed.

## Abbreviations

AUC: area under the curve
eB2: Evidence-Based Behavior
EMA: Ecological Momentary Assessment
FPR: false positive rate
TPR: true positive rate

## Data Availability

Data are available on demand.
